# 

**Published:** 1920-02-07

**Authors:** 


### THE HOSPITAL

**The Modern Newspaper of Administrative Medicine and Institutional Life.**

Telegraphic Address: OSPEDALE, RAND, LONDON. Telephone Number: 2734 GERRARD

No. 1757, Vol. LXVII. | Saturday, February 7th, 1920. | PRICE TWOPENCE

